# Secondary Metabolites of Mangrove-Associated Strains of *Talaromyces*

**DOI:** 10.3390/md16010012

**Published:** 2018-01-06

**Authors:** Rosario Nicoletti, Maria Michela Salvatore, Anna Andolfi

**Affiliations:** 1 Council for Agricultural Research and Agricultural Economy Analysis, 00184 Rome, Italy; rosario.nicoletti@crea.gov.it; 2 Department of Agriculture, University of Naples ‘Federico II’, 80055 Portici, Italy; 3 Department of Chemical Sciences, University of Naples ‘Federico II’, 80125 Naples, Italy; mariamichela.salvatore@unina.it

**Keywords:** bioactive products, drug discovery, endophytic fungi, mangroves, *Talaromyces*

## Abstract

Boosted by the general aim of exploiting the biotechnological potential of the microbial component of biodiversity, research on the secondary metabolite production of endophytic fungi has remarkably increased in the past few decades. Novel compounds and bioactivities have resulted from this work, which has stimulated a more thorough consideration of various natural ecosystems as conducive contexts for the discovery of new drugs. Thriving at the frontier between land and sea, mangrove forests represent one of the most valuable areas in this respect. The present paper offers a review of the research on the characterization and biological activities of secondary metabolites from manglicolous strains of species belonging to the genus *Talaromyces*. Aspects concerning the opportunity for a more reliable identification of this biological material in the light of recent taxonomic revisions are also discussed.

## 1. Introduction

The establishment of the concept of ‘one fungus, one name’ in mycology [1] has stimulated reconsideration of the nomenclature of fungi, whose anamorphic stages were until recently grouped in the genus *Penicillium*, and included species renowned for being among the most prolific producers of bioactive secondary metabolites and a few blockbuster drugs [2,3,4]. In fact, a fundamental taxonomic revision has ultimately established that species with symmetrical biverticillate conidiophores, which were formerly ascribed to the *Penicillium* subgenus *Biverticillium*, are to be classified separately in the genus *Talaromyces*, and that *Penicillium* and *Talaromyces* belong to phylogenetic lineages that are distant enough to deserve ascription to different families [5,6]. Under the ecological viewpoint, recent reports are depicting a widespread endophytic occurrence of *Talaromyces* [7,8,9,10], which makes these fungi increasingly considered a source of interesting bioactive compounds.

After a few years, the above revision has not yet found full consideration. This is particularly true among researchers working in the field of drug discovery, who sometimes do not possess a robust mycological background. In fact, in a number of recent reports limiting identification to the genus level, the name *Penicillium* sp. is still inappropriately used for strains displaying the symmetrical biverticillate conidiophore condition. It is of course desirable that hasty investigators be more circumstantial in considering this fundamental step when reporting on their findings. Moreover, in contrast to the purpose of increasing accuracy, the adoption of identification procedures that are only based on DNA sequence homology has sometimes introduced additional approximation, considering that plenty of sequences referring to ‘*Penicillium* sp.’ have been deposited in GenBank, and are routinely used as a support for the incomplete classification of new strains. Pending the diffusion of more decisive identification protocols, a good portion of the work carried out so far in the field of the purification and characterization of secondary metabolites from *Penicillium*/*Talaromyces* strains awaits revision in order to attain a more conclusive taxonomic ascription of this biological material, and avoid possible confusion from unreliable information. In fact, data concerning the production of secondary metabolites can be quite informative for these fungi, particularly when they are indicative of the ability to synthesize some structural models that are only, or predominantly, found in *Talaromyces* [11,12].

Following a recent paper on bioactive compounds from *Talaromyces* strains obtained from other marine sources [3], this review examines literature concerning secondary metabolites produced by these fungi recovered in association with mangrove plants, including a number of reports adopting the generic denomination ‘*Penicillium* sp.’.

## 2. Mangrove Swamps: A Dynamic Frontier between Land and Sea

Spread along the coastlines at tropical and subtropical latitudes, mangrove forests are a biodiversity hotspot as well as a peculiar transition ecosystem, harboring organisms that are typical of either marine or terrestrial habitats. Considering their prevalently emerged bearing, mangrove plants cannot be considered real marine organisms to the same extent as seagrasses [3]. However, they play a key role in maintaining and building soil from the intertidal zone, and are morphologically and physiologically adapted to the particularly harsh environmental conditions deriving from a combination of extensive salinity, tide alternation, anaerobic clayey soil, high temperature, and moisture [13].

Mangrove plants host a great variety of endophytic and other associated fungi, a good part of which derives from the surrounding soil, marine, and freshwater contexts. Regardless of their true origin, which in most instances cannot be proven, these symbionts might contribute to their host’s adaptation in such a peculiar habitat [14]. According to the plant species, the environmental conditions, and other factors, a wide set of interactions are potentially established between endophytes and their hosts [15]. However, the most considered aspect is represented by the mutual effects on the production of secondary metabolites. Recent investigations have demonstrated that these secondary metabolites are regulated by complex biomolecular mechanisms, such as chromatin methylation [16], and are regarded as fundamental mediators of interspecific communication [17]. In applicative terms, this intriguing ecological scenario reflects a series of bioactive properties of a multitude of structurally diverse compounds that these fungi are able to synthesize, stimulating their consideration as one of the most promising sources for drug prospects [14,18,19,20,21,22].

## 3. The Occurrence of *Talaromyces* Species in Mangroves

In the last decade, literature concerning drug discovery has been substantially enriched by many reports dealing with the biosynthetic potential of mangrove-associated fungi. Also, there has been an increasing trend over the past few years in the finding of *Talaromyces* strains from this particular ecological context, which appears to be in evident connection with its quite recent spread in nomenclatural use following the formal separation from *Penicillium*. However, apart from two cases from South America, these reports all refer to locations in southeast Asia, particularly from the Chinese provinces of Fujian, Guangdong, and Guangxi, and Hainan Island (Table 1).

However, it is questionable whether some of these reports are actually replications. In fact, the strains YX1 and HZ-YX1 obtained from leaf samples of *Kandelia obovata* were claimed to have been collected in April 2012 at two locations in the Guangdong province situated over 400 km apart. Both strains were ascribed to the species *T. amestolkiae* based on rDNA-ITS sequence homology; nevertheless, the same GenBank accession code is indicated by the authors, which refers to Zhanjiang as the place of origin (hence strain YX1) [24,25]. Even more ambiguous is the case of strain 9EB of *T. aculeatus*, whose identification was again based on the homology of a 16S sequence of 576 bp deposited in GenBank (accession code: KT715695), which is actually referred to a strain of *Penicillium* sp. that had been given a different number (C08652) [23]. However, this sequence is identical to one from another strain (CY196, accession number: KP059103) identified as *T. verruculosus*, again submitted from Chinese researchers from Guangzhou. Finally, substantial perplexity arises for three strains labeled with the same number (FJ-1) despite a declared different origin, which are reported to have been identified through rDNA-ITS sequencing [33,34,35]. However, the GenBank code (DQ365947.1) provided for all of them actually corresponds to a previously deposited sequence from a strain of *T. purpurogenus* (HS-A82).

## 4. Structures and Properties of Secondary Metabolites from Manglicolous *Talaromyces*

Most of the strains mentioned in Table 1 were reported for the production/bioactive effects of secondary metabolites, which undoubtedly represent the major objective prompting research on endophytic fungi. The structure of these compounds was essentially elucidated by means of spectroscopic methods, such as two-dimensional (2D) NMR and mass spectrometry. In some cases, their absolute configuration was determined through a modified Mosher’s method or electronic circular dichroism (ECD) spectra, or the structures confirmed by means of single-crystal X-ray diffraction experiments. So far, 39 new compounds out of a total of 88 (Table 2) have resulted from the biochemical characterization of these strains. Aside from a few quite original structural models, most of them are strictly correlated to known products that have been previously reported from other strains of *Talaromyces* [2,3,11]. A lower number of compounds (22) already known from this genus have also been identified in manglicolous strains, indicating that research in this particular field has yielded a notable percentage of new products. However, it is not possible to infer whether these numbers subtend any specific biosynthetic abilities, considering that it is quite likely that a few novel products were not previously detected in strains of different origin by the simple reason that they had not been characterized yet.

The majority of these secondary metabolites have been evaluated for some kind of biological properties, particularly cytotoxic/antiproliferative activity against tumor cell lines, antimicrobial effects against bacterial and fungal strains, and immunosuppressive and enzyme inhibitory aptitudes. However, some interesting effects have been also described for many of the other 49 compounds previously reported from other biological sources, which have not been specifically considered in Table 2.

As a likely result of evolutionary pressure, genes encoding fungal secondary metabolites are known to be clustered, and their synthesis is known to occur through a few common schemes, such as the acetate, shikimate, and mevalonate pathways [42]. Nevertheless, the molecular structure of these compounds is very varied, even within a single genus such as *Talaromyces*, and a convenient discussion should be based on their grouping in different classes [43].

Depsidones are ester-like depsides, or cyclic ethers, which are related to the diphenyl ethers, and synthesized through the polymalonate pathway. Their structure is based on an 11*H*-dibenzo(*b*,*e*) [1,4] dioxepin-11-one ring system where bridging at the phenolic group in the *p*-position can result in increased antioxidant activity. The efficient antioxidant properties of depsidones may also derive from their incorporation into lipid microdomains [44]. Since antioxidant properties are in turn related to anti-inflammatory, antiproliferative, and antiviral activities, compounds from *Talaromyces* spp. belonging to this class, particularly the novel talaromyones A and B [39], should be better investigated with reference to these bioactive effects. Funicones and the related vermistatins probably represent the most typical class of secondary metabolites produced by *Talaromyces* spp., possessing several bioactive properties that make them renowned drug prospects [45]. Particularly, 3-*O*-methylfunicone has displayed notable antifungal, antitumor, and lipid-lowering properties that require more circumstantial investigations beyond academic research, for which a direct support by the pharmaceutical industry seems to be fundamental [46,47,48,49,50,51]. A few novel vermistatin derivatives obtained from a manglicolous strain of *T. pinophilus* have been characterized as α-glucosidase inhibitors [29].

In fungi, both anthraquinones and xanthones are reported to be synthesized through the cyclization of polyacetate units, in the latter case followed by oxidative cleavage of the central ring [52]. Well-known mycotoxins ascribed to these groups, such as emodin, skyrin, secalonic acid A, and norlichexanthone, have been also reported as secondary metabolites of a manglicolous *Talaromyces* strain [38]. The related benzophenones are represented by the new potent immunosuppressive product peniphenone and its methyl derivative [32]. Other phenolic metabolites are possibly synthesized following the shikimate pathway [53], such as two new biphenyl and phenylcyclopentenone derivatives that have been characterized for their α-glucosidase inhibitory effects [41].

Benzofurans, also known as coumarones, represent another important class of natural products, and a scaffold considered for the development of synthetic drugs [54]. This group includes two new compounds derived from strain YX1 of *T. amestolkiae*, which exhibit antibacterial activities [24]. Again known for a wide array of pharmacological properties, isocoumarins are coumarin isomers presenting an inverted lactone ring, most of which possess a 3-alkyl or a 3-phenyl moiety on a α-pyranone nucleus, and 8-oxygenation on the benzene ring. The discovery of novel natural isocoumarins is ongoing; a few hundreds of isocoumarins and dihydroisocoumarins are currently known from different sources [55]. Despite such a high diversity, the number of isocoumarins displaying a completely different substitution pattern is quite reduced, and most of the newly isolated products turn out to be derivatives of previously known structures. A good example is represented by a series of known and novel compounds by the above-mentioned strain YX1, which have been again characterized for their α-glucosidase inhibitory effects [24]. The talaroflavones, including the new antibacterial analogue 7-hydroxy-deoxytalaroflavone [33], are also ascribed to this class.

Azaphilones are a typical class of fungal red or purple pigments with pyrone–quinone structures containing a highly oxygenated bicyclic core and a chiral quaternary center, whose use as colorants has been proposed in several fields, including the food industry [56]. These compounds exhibit a wide range of bioactivities, deriving from antimicrobial, antiviral, antioxidant, cytotoxic, nematicidal, and anti-inflammatory properties [57,58]. New members of this family are represented by the antibacterial/cytotoxic product 7-epiaustdiol and its methyl derivative [38], and the pinazaphilones, which have been characterized as α-glucosidase inhibitors [41]. Another red pigment, glauconic acid, is probably the oldest product mentioned in this review. In fact, this nonadride compound has been known since 1931 [59], mainly from studies concerning its biosynthetic pathway, which indicate that it derives through several steps involving substitutions in citric acid and dimerization of a C_9_ anhydride unit [60], or from succinate [61]. However, no detailed investigation of its bioactivity seems to have been accomplished so far. α-glucosidase inhibitory activity also characterizes bacillosporins (bacillisporins) A and B, two known antibacterial oligophenalenone dimers reported together with a new chromone from a strain of *T. aculeatus* [23]. Additional novel polyketides from manglicolous *Talaromyces* strains are represented by leptosphaerone C, a cytotoxic cyclohexenone derivative [32], and the flavonoid (2*R*,3*S*)-pinobanksin-3-cinnamate, displaying interesting neuroprotective effects [35]. 

Although alkaloids are widespread secondary metabolites of endophytic fungi [62], only two representatives of this class have been reported from mangrove-associated *Talaromyces* strains. Particularly, ZG-1494α is a pyrrolidinone derivative that has been reported as an inhibitor of the platelet-activating factor acetyltransferase [63], while talaramide A is a new compound presenting an unusual oxidized tricyclic system, which has been characterized for its antimycobacterial properties deriving from PknG kinase inhibitory effects [25].

Finally, the terpenes also appear to be quite infrequent from this particular microbial source. They include the sesquiterpene amino acid-alcohol ester purpuride [26], and a few novel cytotoxic-antiproliferative products, namely 15-hydroxy-6α,12-epoxy-7β,10αH,11βH-spiroax-4-ene-12-one [34], 15-α-hydroxy-(22*E*,24*R*)-ergosta-3,5,8(14),22-tetraen-7-one [35], and the talaperoxide series [27].

## 5. Conclusions

The availability of increasingly refined laboratory equipment, and the ability to access previously hindered sources for the isolation of novel fungal strains has stimulated a huge amount of research activity in view of identifying new bioactive compounds and drugs. Moreover, novel accurate screening strategies and procedures have been introduced for a targeted selection in view of reducing the misuse of resources and ensuing replication through finding known compounds [64,65,66,67]. With an increasing rate of recovery from both terrestrial and marine environmental contexts, and a wide range of ecological interactions with other organisms, *Talaromyces* strains are among the most promising ‘biofactories’ that can further enlarge the current panorama of bioactive products available for exploitation by the pharmaceutical industry. Considering the relatively reduced extension of the areas covered by such investigations so far, this remarkable potential deserves to be more thoroughly appreciated, particularly by spreading the search for new strains all over the manglicolous regions that have not yet been considered.

## Figures and Tables

**Table 1:** List of mangrove-associated *Talaromyces* strains gathered from the literature.

Species/Strain	Source	Location	Reference
*T. aculeatus*/9EB	*Kandelia candel* (leaf)	Yangjiang (Guangdong), China	[23]
*T. amestolkiae*/YX1	*Kandelia obovata* (leaf)	Zhanjiang Mangrove Natural Reserve (Guangdong), China	[24]
*T. amestolkiae*/HZ-YX1	*K. obovata* (leaf)	Huizhou Mangrove Natural Reserve (Guangdong), China	[25]
*T. atroroseus*/IBT 20955	*Laguncularia racemosa* (root)	Paria Bay, Venezuela	[26]
*T. flavus*/CCTCCM2010266	*Sonneratia apetala* (leaf)	Hainan, China	[27]
*T. funiculosus*	*Avicennia officinalis* (root) *Rhizophora mucronata* (root) undetermined species (leaf)	Pichavaram (Tamil Nadu), India	[28]
*T. pinophilus*/HN29-3B1	*Cerbera manghas*	Dong Zhai Gang Mangrove Natural Reserve (Hainan), China	[29]
*T. pinophilus*	*Ceriops tagal* (root)	Dong Zhai Gang (Hainan), China	[30]
*T. pinophilus*	*L. racemosa* (leaf)	Itamaracá Island, Brazil	[31]
*T. purpurogenus*/JP-1	*Aegiceras corniculatum* (bark)	Fujian, China	[32]
*Talaromyces* sp./FJ-1 ^1^	*C. tagal* (stem)	Haikou (Hainan), China	[33]
*Talaromyces* sp./FJ-1 ^1^	*Avicennia marina*	Fujian, China	[34]
*Talaromyces* sp./FJ-1 ^1^	*Acanthus ilicifolius*	Hainan, China	[35]
*Talaromyces* sp./ZJ-SY2 ^1^	*S. apetala* (leaf)	Zhanjiang Mangrove Natural Reserve (Guangdong), China	[36]
*Talaromyces* sp./SBE-14	*K. candel* (bark)	Hong Kong, China	[37]
*Talaromyces* sp./ZH154	*K. candel* (bark)	Zhuhai (Guangdong), China	[38]
*T. stipitatus*/SK-4	*A. ilicifolius* (leaf)	Shankou Mangrove Natural Reserve (Guangxi), China	[39]
*T. trachyspermus*/KUFA35	not specified	Thailand	[40]

^1^ These strains reported as *Penicillium* sp.

**Table 2:** Structures and bioactivities of secondary metabolites produced by manglicolous *Talaromyces* strains. The names of novel compounds are underlined. Compounds marked by an asterisk were previously reported from *Talaromyces* strains from sources other than mangroves [3,11,12].

Compound Name	Structure	Reported Bioactivities	Reference
**Depsidones, Diphenyl Ether Derivatives**			
Penicillide * (R = H) Purpactin A * (=vermixocin B) (R = CH_3_CO)	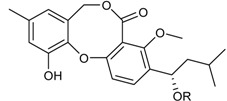		[39]
Secopenicillide B	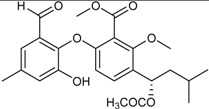		[39]
Talaromyone A (R = H)	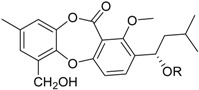	Antibacterial: (MIC μg/mL) *B. subtilis* 12.5 (talaromyone B)	[39]
Talaromyone B (R = CH_3_CO)		α-Glucosidase inhibitor (IC_50_ μM) 48.4 (talaromyone B)	
Tenelate A (R = H) Tenelate B (R = CH_2_CH_3_)	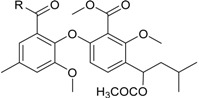		[37]
Tenellic acid A *	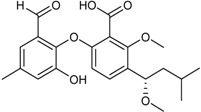	α-Glucosidase inhibitor (IC_50_ μM) 99.8	[39]
Tenellic acid C	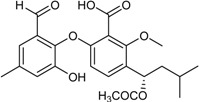		[37,39]
**Funicones, Vermistatins**			
3-*O*-Methylfunicone *	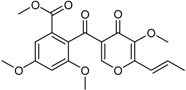		[41]
Penicidone D	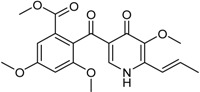		[41]
(±)-Penifupyrone	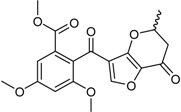	α-Glucosidase inhibitor (IC_50_ μM) 14.4	[41]
Penisimplicissin * (R_1_ = H, R_2_ = CH_3_) 6-Demethylpenisimplicissin (R_1_ = R_2_ = H) 5′-Hydroxypenisimplicissin (R_1_ = OH, R_2_ = CH_3_)	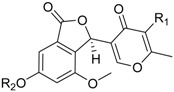	α-Glucosidase inhibitor (IC_50_ μM) 9.5 (6-demethylpenisimplicissin)	[29]
Vermistatin * (R = H) Hydroxyvermistatin * (R = OH) Methoxyvermistatin * (R = OCH_3_)	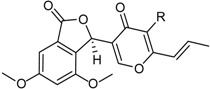	α-Glucosidase inhibitors (IC_50_ μM) 29.2, 20.3 ^1^	[29]
2′′-Epihydroxydihydrovermistatin	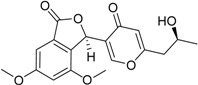	α-Glucosidase inhibitor (IC_50_ μM) 8.0	[29]
6-Demethylvermistatin	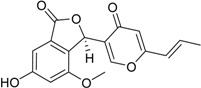		[29]
**Anthraquinones**			
Emodin *	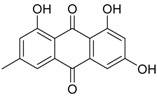	Antibacterial: (MIC μg/mL) *E. coli* 6.25; *P. aeruginosa* 12.5; *S. ventriculi* 12.5; *S. aureus* 12.5	[38]
		Antifungal: (MIC μg/mL) *A. niger* 12.5; *C. albicans* 6.25; *F. oxysporum* f.sp. *cubense* 25.0	
		Cytotoxic: (IC_50_ μg/mL) KB 12.43; KBv200 15.72	
Skyrin *	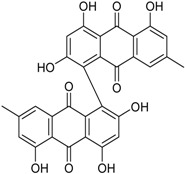	Antibacterial: (MIC μg/mL) *E. coli* 25.0; *P. aeruginosa* 12.5; *S. ventriculi* 25.0; *S. aureus* 25.0	[38]
		Antifungal: (MIC μg/mL) *A. niger* 25.0; *C. albicans* 12.25	
		Cytotoxic: (IC_50_ μg/mL) KB 20.38; KBv200 16.06	
**Xanthones**			
Conioxanthone A (R_1_ = H, R_2_ = R_3_ = OH) 8-Hydroxy-6-methyl-9-oxo-9H-xanthene-1-methylcarboxylate (R_1_ = R_2_ = R_3_ = H) Pinselin (R_1_ = OH, R_2_ = R_3_ = H) Sydowinin A (R_1_ = OH, R_2_ = H, R_3_ = OH) Sydowinin B (R_1_ = R_2_ = H, R_3_ = OH)	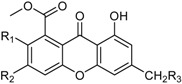	Immunosuppressive: (IC_50_ μg/mL) Con A-Induced 8.2, 25.7, 5.9, 6.5, 19.2 ^1^	[36]
		Immunosuppressive: (IC_50_ μg/mL) LPS-Induced 7.5, 26.4, 7.5, 7.1, 20.8 ^1^	
Norlichexanthone	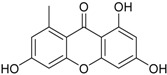	Antibacterial: (MIC μg/mL) *P. aeruginosa* 25.0; *S. ventriculi* 25.0; *S. aureus* 12.5	[38]
		Antifungal (MIC μg/mL) *A. niger* 25.0; *C. albicans* 6.25; *F. oxysporum* f.sp. *cubense* 50.0	
		Cytotoxic: (IC_50_ μg/mL) KB 12.43; KBv200 15.72	
Peniphenone (R = H)	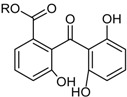	Immunosuppressive: Con A-Induced (IC_50_ μg/mL) 8.1, 17.5 ^1^	[36]
Methylpeniphenone (R = CH_3_)		Immunosuppressive: LPS-Induced (IC_50_ μg/mL) 9.3, 23.7 ^1^	
Remisporine B (R = βH) Epiremisporine B (R = αH)	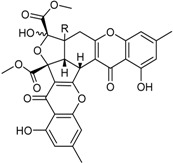		[36]
Secalonic acid A	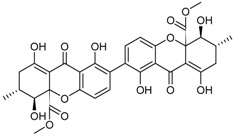	Antibacterial: (MIC μg/mL) *E. coli* 25.0; *P. aeruginosa* 12.5; *S. ventriculi* 12.5; *S. aureus* 12.5	[38]
		Antifungal (MIC μg/mL) *A. niger* 6.25; *C. albicans* 6.25; *F. oxysporum* f.sp. *cubense* 12.5	
		Cytotoxic: (IC_50_ μg/mL) KB 0.63; KBv200 1.05	
Stemphyperylenol	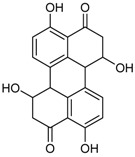	Antibacterial: (MIC μg/mL) *P. aeruginosa* 12.5; *S. ventriculi* 3.12; *S. aureus* 25.0	[38]
		Antifungal: (MIC μg/mL) *A. niger* 50.0; *C. albicans* 6.25	
		Cytotoxic: (IC_50_ μg/mL) KB 20.20; KBv200 44.35	
**Benzophenone Analogs**			
Arugosin I	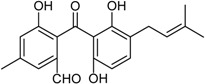		[32]
Penicillenone	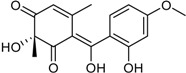	Cytotoxic: (IC_50_ μM) P388 1.38	[32]
**Phenols, Biphenyls**			
4-(2′,3′-Dihydroxy-3′-ethyl-butanoxy)-phenethanol	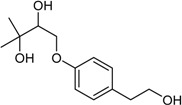	Cytotoxic: (IC_50_ μM) MG-63 35, Tca8113 26	[34]
2,4-Dihydroxy-6-methylbenzoic acid (R = COOH) 5-Methylbenzene-1,3-diol (R = H)	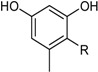		[41]
4′-(*S*)-(3,5-Dihydroxyphenyl)-4′-hydroxy-6′-methylcyclopent-1′-en-5′-one	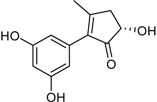		[41]
6′-Methyl-[1,1′-biphenyl]-3,3′,4′,5-tetraol	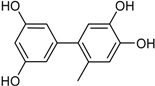	α-Glucosidase inhibitor (IC_50_ μM) 2.2	[41]
**Benzofurans**			
5-Carboxyphthalide	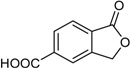		[23]
1-(5-Hydroxy-7-methoxy-benzofuran-3-yl)-ethanone	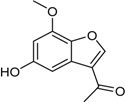	Antibacterial: (MIC μg/mL) *B. subtilis* 50; *E. coli* 50; *S. aureus* 25; *S. epidermidis* 50	[24]
5-Hydroxy-7-methoxy-2-methyl-benzofuran-3-carboxylic acid	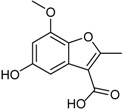	Antibacterial (MIC μg/mL) *B. subtilis* 25; *E. coli* 50; *S. aureus* 25; *S. epidermidis* 25	[24]
**Isocoumarins**			
Aspergillumarin A *	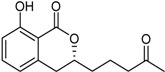	α-Glucosidase inhibitor (IC_50_ μM) 38.1	[24]
Aspergillumarin B * (R_1_ = R_2_ = H) Penicimarin B * (R_1_ = CH_3_, R_2_ = H) Penicimarin C * (R_1_ = CH_3_, R_2_ = OH)	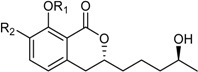	α-Glucosidase inhibitors (IC_50_ μM) 193.1, 431.4, 266.3 ^1^	[24]
6,8-Dihydroxy-3,4-dimethyl-isocoumarin (R_1_ = H, R_2_ = H, R_3_ = CH_3_) 6,8-Dihydroxy-5-methoxy-3-methyl-isochromen-1-one (R_1_ = H, R_2_ = CH_3_, R_3_ = H) 6-Hydroxy-8-methoxy-3,4-dimethylisocoumarin (R_1_ = CH_3_, R_2_ = H, R_3_ = CH_3_)	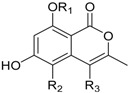	α-Glucosidase inhibitor (IC_50_ μM) 34.4, 89.4, 585.7 ^1^	[24]
3-(4,5-Dihydroxy-pentyl)-8-hydroxy-isochroman-1-one	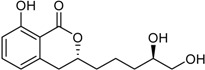	α-Glucosidase inhibitor (IC_50_ μM) 162.5	[24]
5,6-Dihydroxy-3-(4-hydroxy-pentyl)-isochroman-1-one	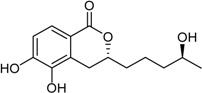	α-Glucosidase inhibitor (IC_50_ μM) 142.1	[24]
6-Hydroxy-4-(1-hydroxy-ethyl)-8-methoxy-isocoumarin ^2^ (R_1_ = CH_3_, R_2_ = H) Sescandelin (R_1_ = R_2_ = H) 5,6,8-Trihydroxy-4-(1-hydroxy-ethyl)-isocoumarin (R_1_ = H, R_2_ = OH)	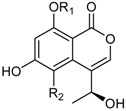	α-Glucosidase inhibitor (IC_50_ μM) 537.3	[24]
6-Hydroxy-4-hydroxymethyl-8-methoxy-3-methyl-isocoumarin (R = CH_3_) Sescandelin B * (R = H)	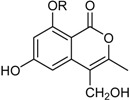	α-Glucosidase inhibitors (IC_50_ μM) 302.6, 17.2 ^1^	[24]
Isobutyric acid 5,7-dihydroxy-2-methyl-4-oxo-3,4-dihydro-naphththalen-1-yl methyl ester	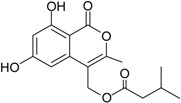	α-Glucosidase inhibitor (IC_50_ μM) 140.8	[24]
Deoxytalaroflavone	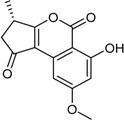	Antibacterial (*S. aureus*)	[33]
7-Hydroxy-deoxytalaroflavone	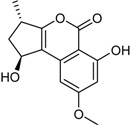	Antibacterial (*S. aureus*, m.r.-*S. aureus*)	[33]
**Azaphilones**			
7-Epiaustdiol (R = H) 8-*O*-Methylepiaustdiol (R = CH_3_)	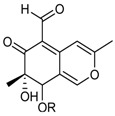	Antibacterial: (MIC μg/mL) *E. coli* > 100, 25; *P. aeruginosa* 6.26, 25.0; *S. ventriculi* 25.0, 50; *S. aureus* 12.6, 50.0 ^1^	[38]
		Antifungal: (MIC μg/mL) *A. niger* 25.0, 50.0; *C. albicans* 12.5, 25.0 ^1^	
		Cytotoxic: (IC_50_ μg/mL) KB 20.04, 16.37; KBv200 19.32, 37.16 ^1^	
Monascorubramine	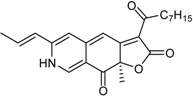		[26]
Monascorubrin	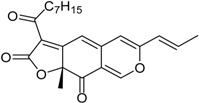		[26]
Pinazaphilone A	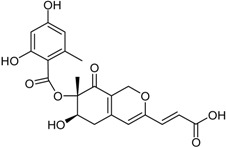	α-Glucosidase inhibitor (IC_50_ μM) 81.7	[41]
Pinazaphilone B (R_1_ = CH_3_, R_2_ = OH) Sch 1385568 * (R_1_ = OH, R_2_ = CH_3_)	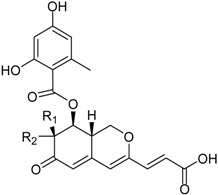	α-Glucosidase inhibitor (IC_50_ μM) 28.0	[41]
Sequoiamonascin D	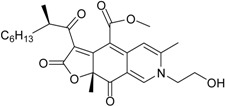		[32]
Sequoiatone A	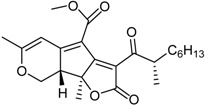		[32]
Sequoiatone B	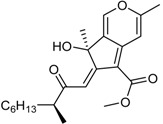		[32]
**Nonadrides**			
Glauconic acid *	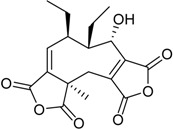		[26]
**Phenalenone Derivatives**			
Bacillosporin A * (R = CH_3_CO) Bacillosporin B * (R = H)	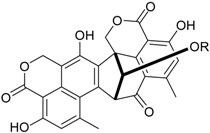	α-Glucosidase inhibitors (IC_50_ μM) 33.55, 95.81 ^1^	[23,32]
Bacillosporin C *	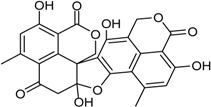		[32]
9-Demethyl FR-901235	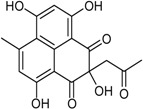		[32]
**Chromones**			
(2′*S* *)-2-(2′-Hydroxypropyl)-5-methyl-7,8-dihydroxy-chromone	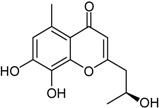	Antibacterial (MIC μM) *Salmonella* 2.0	[23]
**Cyclohexenones**			
Leptosphaerone C	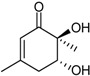	Cytotoxic: (IC_50_ μM) A-549 1.45	[32]
**Flavonoids**			
(2*R*,3*S*)-Pinobanksin-3-cinnamate	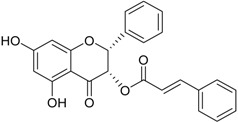	Neuroprotective	[35]
**Alkaloids**			
Talaramide	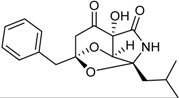	Antimycobacterial: (IC_50_ μM) PknG kinase inhibitor 55	[25]
ZG-1494α *	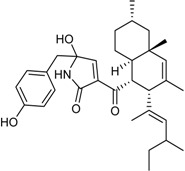		[26]
**Terpenes**			
15-Hydroxy-6α,12-epoxy-7β,10αH,11βH-spiroax-4-ene-12-one	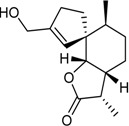	Cytotoxic: (IC_50_ μM) MG-63 55nM, Tca8113 10, WRL-68 58	[34]
15-α-Hydroxy-(22*E*,24*R*)-ergosta-3,5,8(14),22-tetraen-7-one	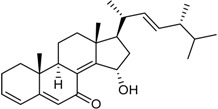	Cytotoxic: glioma cell lines (IC_50_ μM) U251 3.2, BT-325 4.1, SHG-44 2.3	[35]
Purpuride *	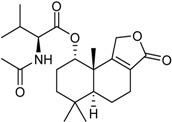		[26]
Steperoxide B (=merulin A) (R = H)	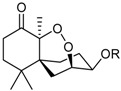	Toxic to brine shrimp	[27]
Talaperoxide A (R = CH_3_CO)		Cytotoxic: (IC_50_ μM) HeLa 7.97, 13.7; HepG2 6.79, 12.93; MCF-7 4.17, 19.77; MDA-MB-435 1.90, 11.78; PC-3 1.82, 5.70 ^1^	
Talaperoxide B	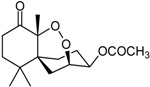	Toxic to brine shrimp Cytotoxic: (IC_50_ μM) HeLa 1.73, HepG2 1.29; MCF-7 1.33; MDA-MB-435 2.78; PC-3 0.89	[27]
Talaperoxide C	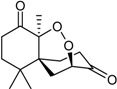	Toxic to brine shrimp Cytotoxic: (IC_50_ μM) HeLa 12.71; HepG2 15.11; MCF-7 6.63; MDA-MB-435 2.64; PC-3 4.34	[27]
Talaperoxide D	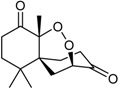	Toxic to brine shrimpCytotoxic: (IC_50_ μM) HeLa 1.31; HepG2 0.90; MCF-7 1.92; MDA-MB-435 0.91; PC-3 0.70	[27]

^1^ Data were reported according to the order of compounds; ^2^ This compound was incorrectly named 5-hydroxy-4-(1-hydroxy-ethyl)-8-methoxy-isocoumarin in the original report.
